# The Glycocalyx and Pressure-Dependent Transcellular Albumin Transport

**DOI:** 10.1007/s13239-020-00489-5

**Published:** 2020-10-01

**Authors:** Randal O. Dull, Andreia Z. Chignalia

**Affiliations:** 1 Department of Anesthesiology, University of Illinois College of Medicine, Chicago, IL USA; 2 Department of Anesthesiology, Banner-University Medical Center, University of Arizona College of Medicine, Suite 4401, 1501 N. Campbell Avenue, Tucson, AZ 85724-5114 USA; 3 Department of Pathology, Banner-University Medical Center, University of Arizona College of Medicine, Tucson, AZ USA; 4 Department of Surgery, Banner-University Medical Center, University of Arizona College of Medicine, Tucson, AZ USA; 5 Department of Physiology, Banner-University Medical Center, University of Arizona College of Medicine, Tucson, AZ USA; 6 Department of Pharmacology and Toxicology, College of Pharmacy, University of Arizona, Tucson, AZ USA

**Keywords:** Caveolea, Endothelium, eNOS, Heparan sulfate, Permeability, Transcytosis

## Abstract

**Purpose:**

Acute increases in hydrostatic pressure activate endothelial signaling pathways that modulate barrier function and vascular permeability. We investigated the role the glycocalyx and established mechanotransduction pathways in pressure-induced albumin transport across rat lung microvascular endothelial cells.

**Methods:**

Rat lung microvascular endothelial cells (RLMEC) were cultured on Costar Snapwell chambers. Cell morphology was assessed using silver nitrate staining. RLMEC were exposed to zero pressure (Control) or 30 cmH_2_O (Pressure) for 30 or 60 min. Intracellular albumin uptake and transcellular albumin transport was quantified. Transcellular transport was reported as solute flux (J_s_) and an effective permeability coefficient (P_e_). The removal of cell surface heparan sulfates (heparinase), inhibition of NOS (L-NAME) and reactive oxygen species (apocynin, Apo) was investigated.

**Results:**

Acute increase in hydrostatic pressure augmented albumin uptake by 30–40% at 60 min and J_s_ and P_e_ both increased significantly. Heparinase increased albumin uptake but attenuated transcellular transport while L-NAME attenuated both pressure-dependent albumin uptake and transport. Apo interrupted albumin uptake under both control and pressure conditions, leading to a near total lack of transcellular transport, suggesting a different mechanism and/or site of action.

**Conclusion:**

Pressure-dependent albumin uptake and transcellular transport is another component of endothelial mechanotransduction and associated regulation of solute flux. This novel albumin uptake and transport pathway is regulated by heparan sulfates and eNOS. Albumin uptake is sensitive to ROS. The physiological and clinical implications of this albumin transport are discussed.

## Introduction

In 1991 Dull *et al.*^6^ published a report characterizing hydraulic conductivity (L_p_), solute flux (J_s_) and the effective permeability coefficient (P_e_) of albumin across cultured aortic endothelial monolayers during step increases in hydrostatic pressures. The relationship of J_s_ and P_e_ versus hydrostatic pressure was non-linear suggesting that pressure altered albumin permeability. At that time, we speculated that pressure-induced convection through the glycocalyx and intra-cellular junction altered the spacing of the fiber matrix such that albumin permeability increased. An alternative explanation was the existence of a pressure-dependent transcytosis mechanism that operated in parallel with paracellular pathways. Over the ensuing 30 years our research efforts on glycocalyx-dependent mechanotransduction and endothelial barrier modulation has provided new significant evidence regarding the effects of pressure on permeability that are mediated by a glycocalyx-endothelial nitric oxide synthase (eNOS) dependent pathway.^4^,^5^,^7^

Kuebler *et al.*^15^ were the first to show that acute increases in lung capillary pressure increased endothelial nitric oxide (NO) and oxidative signaling by a stretch-dependent mechanism. Dull *et al.*^5^ subsequently showed that pressure activated NO production in lung capillary endothelial cells grown on a rigid support where stretch was virtually absent. In those studies, it was shown that pressure-dependent eNOS activation and the increase in L_p_ was mediated by heparan sulfate proteoglycans. Inhibition of eNOS mitigates both pressure and shear-induced changes in permeability as demonstrated using a variety of endothelial cell types.^5^,^11^,^15^–^17^ Dull *et al.*^7^ extend these studies to a perfused lung model and demonstrated that acute increase in pulmonary capillary pressure resulted in an increase in lung filtration coefficient (K_f_) and this was mediated by a heparan sulfate-nitric oxide mechanism. More recently, we demonstrated that inhibition of lung NOS, prevented pulmonary edema in a rat model of acute hypertensive heart failure, despite persistently elevated pulmonary capillary pressure.^4^

## Methods

### Reagents

Krebs–Henseleit buffer, *N*_ω_-nitro-l-arginine methyl ester (L-NAME), apocynin were purchased from Sigma-Aldrich (St Louis, MO). Bovine serum albumin (BSA) was purchased from Proliant (Ankeny, IA). BSA-FITC conjugate was purchased from ThermoFisher Scientific (Waltham, MA). Snapwells were purchased from Corning Life Sciences (Corning, NY). Albumin Blue Fluorescent kit was purchased from Active Motif (Carlsbad, CA). Heparinase III was purchased from New England Biolabs (Ipswich, MA).

### Cell Culture

Rat lung microvascular endothelial cells (RLMEC) were purchased from VEC Technologies (Rensselaer, NY) and cultured with EBM-2 medium (Lonza, Walkersville, MD) supplemented with 10% fetal bovine serum; cells were maintained in an incubator at 37 °C, 5% CO_2_. Cells from passages 4 to 8 were used in these experiments. Cells were serum-deprived in Krebs–Henseilet buffer supplemented with 3% BSA for 2 h before exposure to hydrostatic pressure (0 or 30 cm of water pressure; low and high pressure respectively) for 30 to 60 min.

RLMEC (passage 4–8) were seeded onto a polycarbonate filter (Transwell chambers, 12 mm diameter, 0.4-*µ*m pore size, 1.12 cm^2^ growth area) which had been pretreated with 0.4% gelatin and 20 *µ*g/mL fibronectin). Cells were cultured for 8 days in EBM-2 medium supplemented with 10% FBS.

### Silver Nitrate Staining

Since transport studies mandate a confluent monolayer, we assessed confluency and morphology by silver nitrate staining. Cells were rinsed in ice-cold PBS and stained with silver nitrate as follows: Transwells were immersed on 5% glucose in water 30 s, 2.5% Silver nitrate for 60 s rinsed in 5% glucose. Cells were then incubated in 1% ammonium bromide for 60 s in water and rinsed in 5% glucose. Cells were fixed in 4% paraformaldehyde and membranes from Transwell were cut and mounted using Permount^®^ mounting media. Cells were visualized on Zeiss microscope and images were acquired using Zen Software (Zeiss).

### Pressure Chamber and Albumin Uptake

The pressure chamber consisted of a polycarbonate block containing six wells fitted with o-rings to seal against the bottom of Transwell culture insert rim. The wells were filled with cell culture media such that the media just contacted the bottom of the filter containing the endothelial monolayer. The top piece of the pressure chamber was a polycarbonate bock also containing o-rings that sealed against the top side of the Transwell insert rim, thus, a water tight seal was created between the top and bottom of the Transwell chamber. The top piece of the pressure chamber contained six ports for that attached to six separate pressure reservoirs, 1 for each well. The hydrostatic pressure gradient was set by raising the reservoirs to a specified distance above the monolayer. The bottom well contained a sampling port that was open to atmosphere and allowed constant pressure in the lower chamber. The pressure chamber was placed into a water bath set at 37 °C.

Cells were serum deprived for 2 h and then exposed to either no pressure or high pressure (30 cmH_2_O) for 60 min in Krebs–Henseleit buffer supplemented with 3% BSA. Cells were washed three times with ice-cold PBS and cells were lysed with RIPA buffer. Cytosolic proteins were extracted by centrifugation (13,000 rpm, 4 °C, 15 min). Albumin concentration in cytosolic fraction was determined by a fluorimetric plate assay kit (Albumin Blue Fluorescent Kit^®^, Active Motif) according to manufacturer’s instructions. Results are expressed in *µ*g/mL.

### Solute Flux (J_s_) and Effective Permeability Coefficient (P_e_)

After exposure to pressure, 100 *µ*L of the abluminal buffer was collected and fluorescence at 488 nm was measured in a plate reader. A standard curve for BSA (0.3–10 *µ*g/mL) was performed in each experiment. Albumin concentration was determined using the linear regression against the standard curve. J_s_ and P_e_ were calculated using the equations:

$$ {\text{J}}_{\text{s}} = \left( {\Delta {\text{C}}/\Delta {\text{t}}} \right)\left( {\text{V}} \right)/{\text{A}}\;{\text{and}}\;{\text{P}}_{\text{e}} = {\text{J}}_{\text{s}} /{\text{C}}_{\text{p}}, $$

where ΔC/Δt is the change in abluminal FITC-BSA concentration per unit time; V is the volume of medium in the abluminal chamber and A is the area of the monolayer. C_p_ is the luminal concentration of FITC-BSA.

### Statistical Analysis

Data are presented as mean ± SD. Groups were compared using 1-way ANOVA, 2-way ANOVA or Student t test, as appropriate. Tukey post-hoc test was used to compensate for multiple testing procedures. *p* < 0.05 was considered statistically significant.

## Results

### Monolayer Assessment with Silver Nitrate Staining

**Figure 1:**
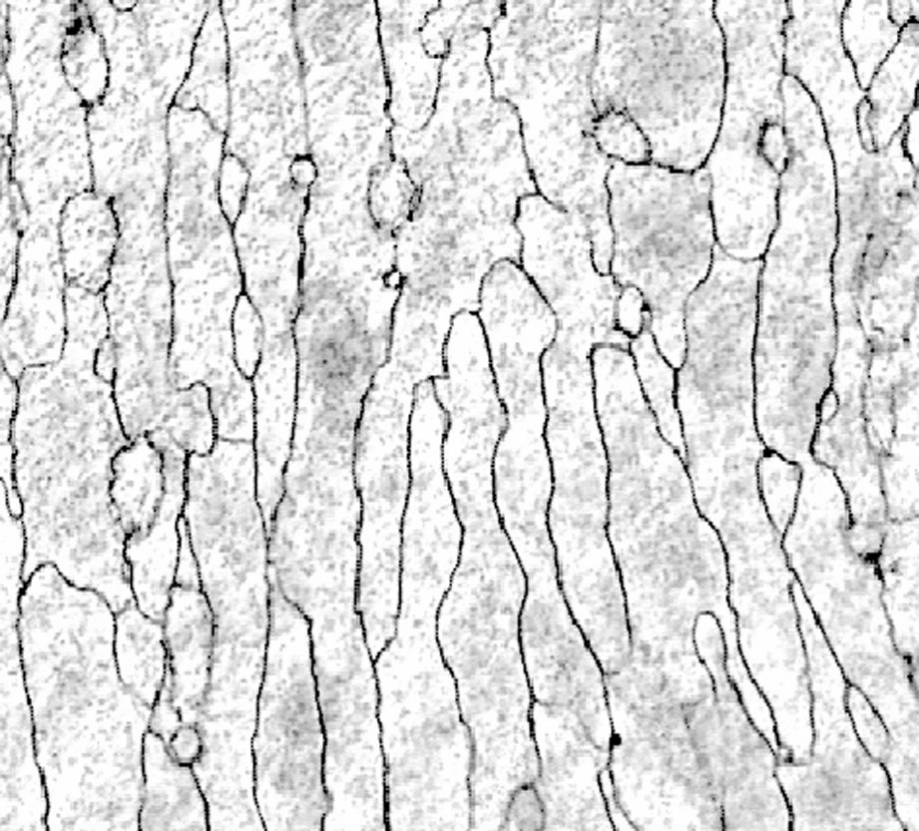
Silver nitrate staining. Representative example of RLMEC monolayer morphology on a polycarbonate filter. RLMEC show complete confluence and typical morphology.

To demonstrate that RLMEC form complete monolayers on polycarbonate filters, we used a standard silver nitrate staining to identify cell membranes. As can be seen in Fig. 1, cells morphology is normal and confluence was found to be 100% across multiple representative samples.

### Albumin Uptake

**Figure 2:**
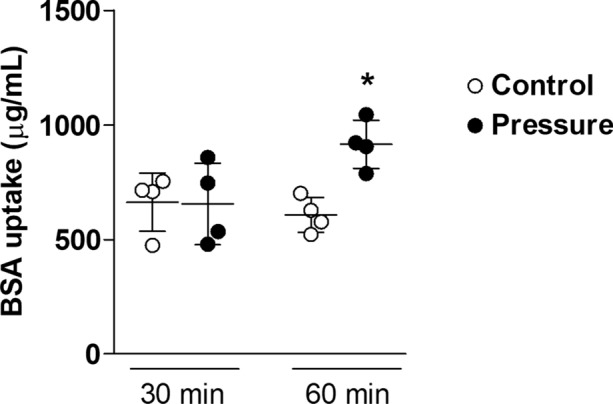
Albumin uptake is both pressure and time dependent. RLMEC albumin uptake was similar in Controls (0 cmH_2_O) vs. Pressure (30 cmH_2_O) after 30 min. Albumin uptake increased by approximately 40% after 60 min in Pressure treated monolayers only. N = 4/group. *p < 0.05 vs. controls at same time (2-way ANOVA, Tukey post hoc test).

Pressure-induced albumin uptake was time-dependent. RLMEC exposed to 30 cmH_2_O for 30 min showed no measurable change in BSA uptake. When monolayers were exposed to 30 cmH_2_O for 60 min albumin uptake increased by 30–40% (Fig. 2).

Basal albumin uptake did not change during the experimental period. In absolute values, control and pressure-treated monolayers had an uptake of 666.2 ± 127.4 and 654.7 ± 177.7 *µ*g/mL at 30 min. At 60 min, BSA uptake increased with pressure as the control monolayers had 607.1 ± 76.1 *µ*g/mL and pressure-treated monolayer increased to 914.9 ± 105.2 *µ*g/mL (p<0.05).

**Figure 3:**
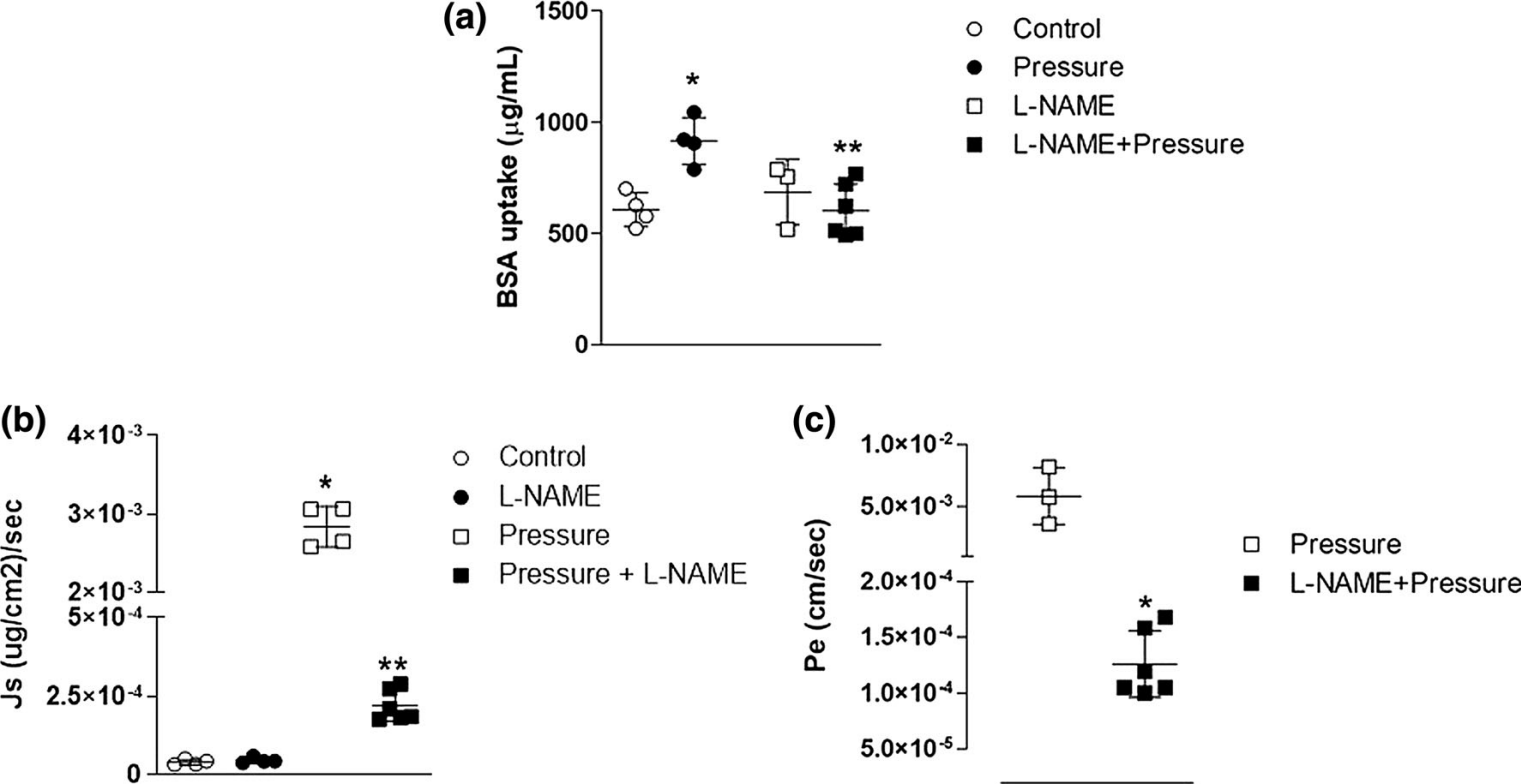
L-NAME attenuates pressure-dependent albumin uptake and transport. (a) Inhibition of NOS by L-NAME completely attenuated RLMEC albumin uptake during exposure to 30 cmH_2_O for 60 min. (b) L-NAME completely prevented solute flux (J_s_) of albumin into the abluminal chamber in RLMEC exposed to 30 cmH_2_O for 60 min. (c) L-NAME significantly reduced the effective permeability coefficient (P_e_) for albumin during exposure to 30 cmH_2_O for 60 min. N = 3–5/group. *, **p < 0.05 vs. control and pressure, respectively (1-way ANOVA, Tukey post hoc used for BSA uptake and J_s_; t test used for Pe).

We previously demonstrated that pressure-dependent changes in endothelial permeability were associated with eNOS activation; therefore, to determine if a similar mechanism was involved, we tested effect of L-NAME (NOS inhibitor) on albumin uptake. The presence of L-NAME (100 *µ*mol/L) completely attenuated pressure-dependent albumin uptake (Pressure + LNAME = 602.7 ± 120.1 *µ*g/mL; Fig. 3). Note that L-NAME did not alter BSA uptake at zero pressure (686.5 ± 147.0 *µ*g/mL), but completely prevented the increase of intracellular BSA content following exposure to pressure of 30 cm H_2_O for 60 min (Fig. 3a).

The L-NAME-dependent reduction of intracellular albumin content could have resulted from either reduced uptake into the cell or enhanced transport of BSA out of the cell. To distinguish which process accounted for the reduced intracellular BSA concentration, we measured albumin transport into the abluminal compartment.

Solute flux (J_s_) was essentially zero across control monolayers incubated with or without L-NAME. J_s_ was completely attenuated at 30 cmH_2_O when L-NAME was present (Table 1, Fig. 3b). The effective permeability coefficient for albumin (P_e_) was significantly reduced by L-NAME (Table 2; Fig. 3c).

**Table 1:** Effect of pressure and inhibitors on solute flux (Js).

Group	Js [(*µ*g/cm^2^)/s] × 10^−5^	SD (×10^−6^)
Control	3.9	8.7
Pressure	284.1*	26.0
L-NAME	4.5*^&^	9.6
L-NAME + pressure	21.9^&^	49.3
Heparinase	ND	ND
Heparinase + pressure	29.0^&^	101.8
Apocynin	3.62	6.8
Apocynin + pressure	8.9^&^	25.1

Representative values of Js in different experimental groups

SD = standard deviation. *p<0.05 vs. control and ^&^p<0.05 vs pressure. *N* ≥ 3/group. Anova one-way, Tukey post hoc test

**Table 2:** Effect of pressure and inhibitors on effective permeability coefficient (Pe).

Group	Pe [Js/Cp] × 10^−3^	SD (10^−5^)
Pressure	5.0	22.2
L-NAME + pressure	0.12*	29.7
Heparinase + pressure	0.24*	19.0
Apocynin + pressure	0.10*	2.24

Representative values of Pe in different experimental groups

*p<0.05. Pressure. SD = standard deviation. N = 3–6/group

## Effect of Heparinase on BSA Uptake and Transport

**Figure 4:**
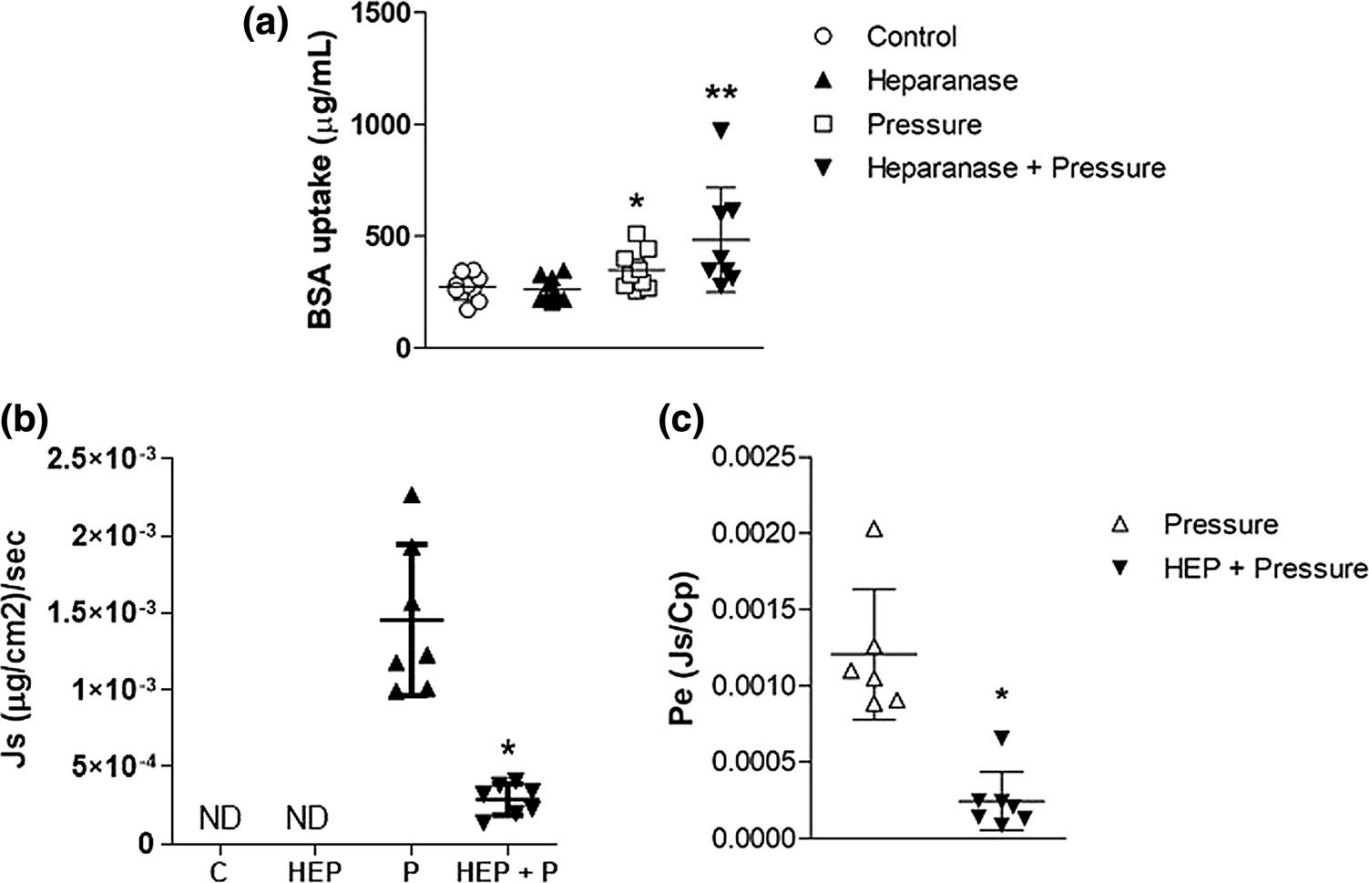
Heparinase attenuates pressure-dependent albumin transport. (a) Heparinase had no effect on RLMEC albumin uptake during control conditions. Heparinase significantly increased cellular uptake of albumin during pressure of 30 cmH_2_O for 60 min. (b) Heparinase significantly reduced the solute flux (J_s_) of albumin across RLMEC during exposure to 30 cmH_2_O for 60 min. (c) Heparinase significantly reduced the effective permeability coefficient (P_e_) for albumin across RLMEC during exposure to 30 cmH_2_O for 60 min. N≥9/group. *, ** p < 0.05 vs. control and pressure, respectively. (1-way ANOVA, Tukey post hoc used for BSA uptake and J_s_; *t*-test used for P_e_).

Pressure-dependent eNOS activation occurs *via* a heparan-sulfate dependent mechanism.^5^,^7^ Therefore, we tested the effect of removal of cell-surface heparan sulfates on pressure-dependent albumin uptake. The removal of cell-surface heparan sulfates after incubation of RLMEC with heparinase had no effect on albumin uptake in the control group (control = 272.9 ± 58.6 vs. heparinase 260.1 ± 53.3 *µ*g/mL) but significantly increased pressure-dependent albumin uptake (pressure = 348.3 ± 78.1 vs. pressure + heparinase (482.8 ± 234.7 *µ*g/mL) (Fig. 4a).

Despite the increase in BSA uptake, heparinase interfered with albumin transport by significantly reducing both J_s_ and P_e_ (Table 1 and 2, Figs. 4b and 4c).

## Effect of Reactive Oxygen Species on BSA Uptake and Transport

**Figure 5:**
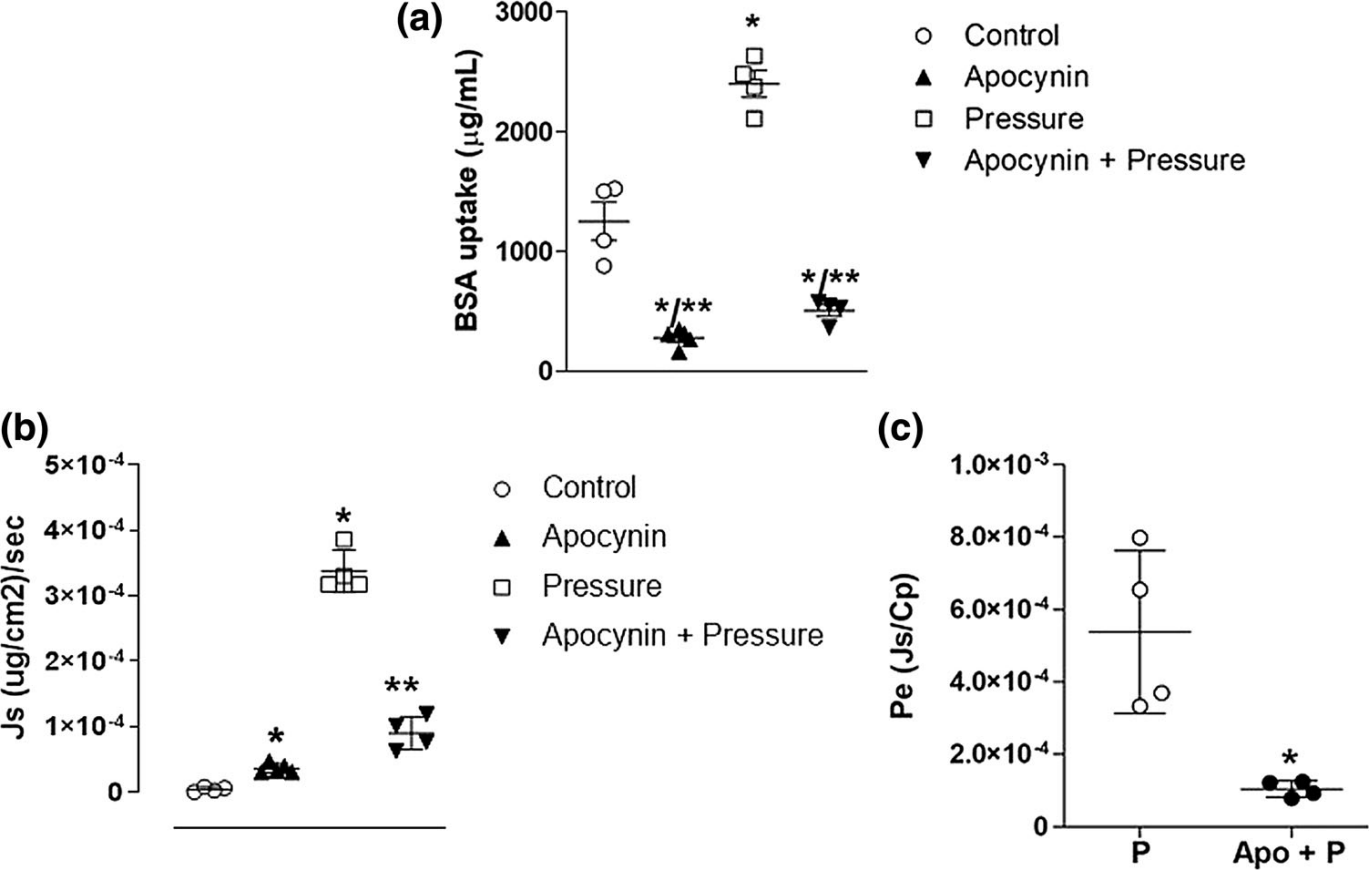
Apocynin reduced basal and pressure-dependent albumin uptake and transport. (a) Apocynin (Apo) reduced albumin uptake in control monolayers (C vs. Apo) at 0 cmH_2_O and reduced albumin uptake in pressure-treated monolayers (P vs. P+ Apo) exposed to 30 cmH_2_O for 60 min. (b) Apo increased albumin solute flux (Js) during basal conditions but significanty decreased J_s_ during high pressure conditions (30 cmH_2_O for 60 min). (c) Apo significantly reduced the effective permeability coefficient (P_e_) during high pressure conditions (30 cmH_2_O for 60 min). N = 4–5/group. *,**p < 0.05 vs. control and pressure, respectively. (1-way ANOVA, Tukey post hoc used for BSA uptake and J_s_; *t*-test used for P_e_).

We then assessed the contribution of reactive oxygen species on albumin uptake and transport using apocynin (Apo), an anti-oxidant. During low pressure conditions, Apo reduced albumin uptake to about 50% of basal levels (279.2 ± 70.76 *µ*g/mL). When Apo was present during exposure to 30 cmH_2_O for 60 min, albumin uptake was nearly abolished (504.4 ± 95.63 *µ*g/mL Fig. 5a). Apo significantly reduced J_s_ and P_e_ during high pressure (Tables 1 and 2, respectively; Figs. 5b and 5c).

## Discussion

The results of these studies demonstrate the existence of a novel pressure-dependent albumin uptake and transcellular transport mechanisms that is mediated, in part, by a heparan sulfate-eNOS-dependent pathway. This process is pressure and time dependent, as 30 cmH_2_O required a duration of 60 min to increase both albumin uptake and transport.

It is unlikely that stretch of the polycarbonate filter and endothelial monolayer played any role in the mechano-transduction processes investigated in this study. The filters on the Snapwell culture insert are very tight and the low hydrostatic pressure gradient (10 and 30 cmH_2_O) is insufficient to stretch the membrane. In fact, Tarbell *et al.*^17^ tested this very issue. To prevent stretch of the membrane during the application of the hydrostatic pressure (pressure = 30 cmH_2_O), they placed a rigid screen underneath the polycarbonate membrane that prevented filter distension. There was no difference in the pressure–L_p_ relationship derived from endothelial monolayers on unsupported vs. supported filters. Therefore, these results suggest that pressure is the primary stimulus.

The time dependency of albumin transport is different than changes in water permeability that we have previously observed. Using bovine lung microvascular endothelial monolayers, hydraulic conductivity (L_p_) began to increase with 10–15 min of the pressure increase.^9^ Similar results were observed in K_f_ when using the rat whole lung preparation, where K_f_ increased within 20 min of the onset of pressure elevation.^3^,^7^ In a rat model of acute heart failure, elevation of pulmonary capillary pressure resulted in an acute reduction in arterial oxygen partial pressure within 30 min.^4^ Krishnamoorthy and colleagues^14^ reported a similar time frame for pulmonary failure in the setting of acute hypertension and Kim *et al.*,^13^ studying rat mesenteric microvessels, reported that pressure increased microvascular within 20–30 min. Collectively, these results demonstrate that endothelial mechanotransduction and changes in junctional permeability occur quite quickly.

The activation of this novel pressure-dependent albumin transport pathway bears many similarities to the heparan sulfate-dependent mechanotransduction that we have previously reported on using a variety of models including *in vitro* endothelial monolayers,^5^,^6^ the isolated perfused rat whole-lung preparation^7^ and a rat model of acute heart failure.^4^ In these previous studies, an acute increase in hydrostatic pressure is sensed and transduced by cell-surface heparan sulfate proteoglycans, resulting in activation of eNOS and by an increase in intracellular NO and ROS. The increase in intracellular NO results in nitrosylation of VE cadherin leading to internalization and junctional complex instability.^8^,^10^ The disruption of the adherens junction complex results in barrier failure and increased paracellular permeability. During prolonged pressure elevation, eNOS becomes uncoupled and produces ROS that are also deleterious to barrier function.^4^

Our findings suggest that albumin uptake is also an ROS-dependent process. It is well know that ROS are second messengers that contribute to cell and tissue homeostasis although the role of ROS on albumin uptake is poorly understood. In lung endothelial cells, different sources of ROS can, in basal states, participate in albumin uptake. Potential ROS sources are: NADPH oxidase (Nox), xanthine oxidase and the electron transport chain in the mitochondria. Nox2, the main isoform expressed in endothelial cells, is located in cellular membranes. Thus, Nox2 is in close proximity to the caveolae and other proteins known to regulate albumin transport. Moreover, Nox has been associated to transcytosis induced by c-reactive protein.^2^ Altogether, these suggest that Nox is a likely a regulator of albumin transport in endothelial cells.

Although increased hydrostatic pressure is known to uncouple eNOS in whole lung preparations, this mechanism does not seem to play a role in albumin uptake as apocynin significantly reduces albumin uptake in basal conditions. The effect of Apo during increased pressure is challenging to clearly interpret. By 60 min, previous studies from our laboratory have shown that inhibition of eNOS and reduction in ROS attenuates changes in endothelial permeability.^5^ If albumin uptake is completely blocked by Apo during both control and pressure conditions, then we would expect both J_s_ and P_e_ to reduced since there is not intracellular albumin to transport. Future studies will have to assess other solutes carried by vesiculars mechanism(s) for more fully understand the effect of ROS on pressure-dependent transport.

Albumin transport is presumably mediated by caveolae and their role in enhanced albumin transport has been well characterized. Nitric oxide-dependent activation of *Src* kinase results in caveolin-1 phosphorylation, destabilization of the caveolin-1 oligomers encasing the caveolea which allows the caveolae mouth to open and the vesicle to swell. Albumin enters the caveolea and binds to albumin-binding protein (gp60); albumin can also be transported in the fluid phase. Ultimately, the caveolea neck closes through a dynamic-dependent process and the vesicle is released from lumenal membrane. Cytoskeletal-dependent transport of the caveolea to the abluminal membrane occurs and, upon fusion with the membrane, the vesicular contents are released.^12^

We hypothesized that the pressure-dependent increase in endothelial permeability is an adaptive mechanism for cells to respond to unfavorable mechanical forces. For example, increased blood flow results in increased wall shear-stress that activates eNOS to induce vasodilation and thereby reduce shear-stress to an acceptable level. The endothelial response to shear stress and pressure are very similar: both occur, in part, *via* a heparan sulfate dependent activation of eNOS resulting in increased NO.

In blood vessels that possess smooth muscle cells, the production of NO and subsequent vasodilation maybe enough to decrease the local hydrostatic pressure. In the microcirculation, like lung alveolar capillaries and small venules, however, smooth muscle cells are not present and microvessels may have developed additional compensatory mechanism(s) to reduce pressure. We hypothesize that the early increase in water permeability and later activation of albumin transport reduces intravascular pressure by decreasing intravascular volume.

### Clinical Implications

The present results suggest an added functional dimension to endothelial mechanotransduction: sustained hydrostatic pressure activates an albumin transport mechanism that will increase interstitial colloid osmotic pressure (COP) that will enhance fluid flux across an already leaky endothelium and reduce intravascular pressure more rapidly. Temporally, the processes of pressure-dependent mechanotransduction invoked to reduce hydrostatic pressure occurs in the order: vasodilation (seconds), increased paracellular permeability (10–20 min), activation of transcellular albumin transport (60 min) and, finally, capillary stress failure.^4^ Interestingly, stress failure of endothelial cells during heart failure and high-altitude pulmonary edema appears to be a component of mechanotransduction. Bhattacharya^1^ reviewed some of the evidence to suggest it is a regulated process. Chignalia *et al.*^4^ showed that L-NAME reduced alveolar hemorrhage in a rat model of acute heart failure despite any effect on pulmonary vascular pressure suggesting that stress failure is part of the continuum of pressure-dependent mechanotransduction. Collectively, the temporal effects of pressure on lung capillary permeability including paracellular leak, vesicular transport and endothelial stress failure, and highlights signaling pathways for developing novel heart failure therapies.
